# Tooth Survival Following Non-Surgical Root Canal Treatment in South Korean Adult Population: A 11-Year Follow-Up Study of a Historical Cohort

**DOI:** 10.14744/eej.2021.86648

**Published:** 2022-03-21

**Authors:** Sun-Mi KIM, Eunsuk AHN

**Affiliations:** From the Department of Dental Hygiene (S.M.K.), Wonkwang Health Science University, Jeollabuk-do, South Korea; Division of Climate Change and Health Protection (E.A. 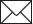 esann82@gmail.com), Korea Disease Control and Prevention Agency, Chungcheongbuk-do, South Korea

**Keywords:** Cohort study, epidemiology, population, root canal treatment (RCT), treatment outcome

## Abstract

**Objective:**

This study examined the survival rate of root canal treatment (RCT) and identified the factors affecting the survival/failure of RCT with respect to the patient’s demographic, socioeconomic and dental healthcare factors.

**Methods:**

The data of patients with RCT were analyzed using the 2002 patient data of the Korean National Health Insurance Service (KNHIS). The analysis included 1,193,666 patients, with 1,414,715 targeted teeth. Survival analysis was performed using the Kaplan-Meier method based on the occurrence of the untoward event. The proportional hazard of failure of RCT was measured using the Cox proportional hazard model and considering variables were gender, age, income, type of dental healthcare, number of visits for RCT, and type of teeth.

**Results:**

The 11-year cumulative survival rate for non-surgical RCT teeth was 88.37%. The Cox proportional hazard model showed significantly lower females (HR 0.704; CI 1.022-1.079) than males. The hazard ratio (HR) of over 65 years (HR 2.959; CI 2.864-3.058) was higher than that of other groups. In addition, the HR varied according to the income level (medical beneficiary was the highest) and the type of dental healthcare (tertiary hospital was the lowest).

**Conclusion:**

Performing RCT survival analysis using representative data revealed that the demographic and socioeconomic factors of the patients affect the failure of RCT. This study can serve as the basis for improving the survival trend in RCT and provide important implications in clinical decision-making in endodontics.

**HIGHLIGHTS**

• The 11-year cumulative survival rate for a tooth with RCT using cohort data was 88.37%.
• The factors affecting the survival rate of teeth undergoing RCT were gender, age, income, health insurance subscription type, number of visits, and tooth type.
• Findings from representative data can help clinicians in decision-making and customizing treatment plans.

## INTRODUCTION

Pulp and apical diseases have high prevalence, and root canal treatment (RCT) is an effective method for maintaining natural teeth (1, 2). RCT primarily prevents reinfection by disinfecting, cleaning, and filling the infected root canal so that the function of the natural teeth in the oral cavity can be maintained (3, 4). However, it is known that microorganisms cannot be completely removed from the root canal due to the limitations of biofilm, bacterial resilience, and chemo-mechanical preparation of the canal for the removal of necrotic tissue (2, 5). Nevertheless, several studies reported that the success rate of RCT is 85-97% (1-7). It is important to make an accurate diagnosis and find the cause of the success or failure of RCT to maintain a consistently high success rate (1, 4). However, studies estimating the survival rate of RCT have shown that different results may be produced depending on various factors such as the study method, treatment procedure, and patient factor (7, 8). Thus, there is insufficient understanding of the criteria for RCT failure and factors affecting the survival rate of RCT (2-5). Hence, it is necessary to adopt an evidence-based approach (5, 6).

In the previous studies, the factors affecting the survival rate of RCT have been divided into demographic and clinical factors. There are significant differences in the clinical factors depending on the clinical procedure (5), final coronal restoration (5-7), presence of apical lesions (6), and the technique used (4, 9). The demographic factors were gender (7, 9), age (10), and income (9, 10), education (11), and occupation (12). Unlike the success rate, the survival rate may not accurately reflect the prognosis of RCT. However, the results of various variables can be compared in epidemiologic studies (13-16). Pineda et al. (12) reported survival rates of 92.3%, focusing on the records of patients treated in Medellin at a Colombian endodontic treatment centre. Salehrabi and Rotstein (17) estimated that the survival rate of RCT was 97% using data from a cohort of patients in 50 states in the US, with most of the teeth with failed RCT requiring additional treatment within three years. In addition,85% of the extracted teeth were not covered with restorations, and there was a significant difference between the groups with and without restorations. A study on the survival rate using data from the Swedish Social Insurance Agency revealed that 25,228 teeth were extracted with a survival rate of 89.8% (18).

Among the previous studies estimating the survival rate of RCT, most studies have used clinical symptoms based on a limited amount of clinical data. However, the use of representative data to examine the survival rate of RCT focusing on patient factors in non-surgical RCT is very rare. Therefore, this study was conducted to confirm the difference in the survival rate of RCT according to patient demographic, socioeconomic, dental healthcare factors. This analysis was conducted using cohort data of 11 years requested from the Korean National Health Insurance Service (KNHIS) from 2002 to 2013.

## MATERIALS AND METHODS

### Data and Variables

The KNHIS is a single insurance that insures the entire nation. It has established a National Health Insurance Database (NHID) of 350.3 billion data points, including qualifications and insurance premiums, health check-up results, and medical history of all citizens based on 2,700 billion original data points. This study was customized to extract only the necessary data from the NHID. Based on the Korean Standard Classification of Diseases (KCD), all patients aged 20 years or older who visited a dental healthcare institution for pulpitis (K04.0) in 2002 were included. The claim data of the patients were followed up from 2002 to 2013. Based on previous studies (17, 19, 20), teeth that underwent RCT procedure and its steps (i.e., access cavity preparation, pulp extirpation, cleaning and shaping, root canal enlargement, and obturation) were targeted. To analyse the survival rate of RCT, it is important to establish criteria for the start and endpoints. Therefore, the start of the survival analysis was set to the time when the treatment codes related to root canal filling (i.e., single-visit endodontics [U0074], root canal filling with single cone method [U0121], and Root canal filling with condensation method [U0126]) were provided. As the survival period was set as 11 years (2002-2013), the end of the survival analysis was set as the time point when the classification code for each treatment activity related to an untoward event was requested (such as anterior tooth extraction [U4412], posterior tooth extraction [U4413], and difficult tooth extraction [U4414], retreatment [U2245], apical resection [U4591, U4592]). The survival endpoint was set as the date of the untoward event of the tooth from the date of RCT in 2002. Therefore, this study looked at the teeth as a standard, and 1,414,715 teeth of 1,193,666 people who underwent RCT in 2002 were finally included. In addition, duplicate cases due to claim data, patients under 20 years of age who underwent RCT, missing income values, missing tooth extraction period, infants, and patients who underwent three or more RCT during one visit in 2002 were excluded from this study. This study was conducted with the approval of the Institutional Review Board (WKIRB-201510-SB-039).

### Study variables

The claim data of the NHIS were personal-based health care utilization data built on an individual basis. Study variables used from the claim data were a reference to the previous study (12, 18), gender (male/female), and age (20-29/30-49/50-64/65 years or older). Insurance types were divided into employee, local subscribers, medical beneficiaries. Health insurance employees received a flat rate based on their monthly income, while local health insurance subscribers claimed it was based on their monthly income and assets. In this study, the income variable was divided into five divisions, and medical beneficiaries were classified as the lowest income. In addition, the type of medical institution (local clinic/hospital/tertiary hospital), number of visits for RCT (multiple visits for treatment, single-visit for treatment), and type of tooth (mandibular molar, mandibular premolar, mandibular anteriors, maxillary molar, maxillary premolar, maxillary anteriors) that underwent RCT. As the dependent variables of this study, an untoward event occurring after RCT, a variable of failure within the follow-up period (failure=1), and survival variable (survival=0) were created, and the period was calculated in months. An 11-year follow-up period from January 1, 2002– December 31, 2013, which is the time period with the maximum data that was provided by the NHIS, was considered. Therefore, depending on the patient, a minimum of 1 and a maximum of 131 months could be followed and investigated.

### Analysis method

In this study, the survival function of the data without the independent variable was estimated using the Kaplan-Meier (K-M) method, which did not provide information on the basis function of the dependent variable and considered the characteristics of the clinical data truncated to the right. Factors influencing the survival rate of RCT were confirmed using the multivariate Cox regression test. All analyses of the study were carried out with the STATA ver. 11.0 (Stata Corp., College Station, TX, USA), the statistical significance was assumed with a p-value <0.05.

## RESULTS

### General characteristics of study participants

This study included 1,414,715 teeth from 1,193,666 individuals who completed RCT and were enrolled in the KNHIS from January 1, 2002 to December 31, 2002. Table 1 presents the general characteristics of the study participants, of whom 53.38% were female and 46.62% were male; 48.04% of the participants were 30-49 years old, the age range with the highest propor-tion. Regarding the type of medical institution, local clinics accounted for 97.47%, and income was highest in the categories with 29.90% (Table 1). As for the tooth types that were successful in root canal treatment, the mandibular molars 28.36%, the maxillary molars 26.79%, and the maxillary premolars 15.10%, were the highest in the order (Table 2).

**TABLE 1.** General characteristics of the study participants (n=1,193,666)

Classification	Values	Number	Percentage (%)
Gender			
	Male	556,459	46.62
	Female	637,207	53.38
Age	20-29	190,383	15.95
	30-49	573,421	48.04
	50-64	304,323	25.49
	≧65	125,539	10.52
Type of institution	Local clinic	1,163,423	97.47
	Hospital	22,078	1.85
	Tertiary Hospital	8,165	0.68
Health insurance subscription type	Local subscribers	735,707	61.63
	Employee	448,641	37.59
	Medical beneficiary	9,318	0.78
Household income categories	Highest	356,914	29.90
	High	276,121	23.13
	High-middle	216,161	18.11
	Low-middle	184,367	15.45
	Low	150,785	12.63
	Lowest (Medical beneficiary)	9,318	0.78

**TABLE 2.** Survival and failure by dental factors (n=1,414,715)

Classification	Values	Survival (n=1,250,285) n (%)	Failure (n=164,430) n (%)
Number of visits	Multiple visits for treatment	1,231,013 (98.45)	162,372 (98.74)
	Single-visit for treatment	19,272 (1.54)	2,058 (1.35)
Tooth type	Maxillary anteriors	158,108 (12.64)	13,801 (8.39)
	Maxillary premolar	188,796 (15.10)	20,532 (12.48)
	Maxillary molar	335,019 (26.79)	52,781 (32.09)
	Mandibular anteriors	63,509 (5.07)	5,174 (3.14)
	Mandibular premolar	150,191 (12.01)	13,320 (8.10)
	Mandibular molar	354,662 (28.36)	58,822 (35.77)

### Kaplan-Meier curves

**Figure 1.**
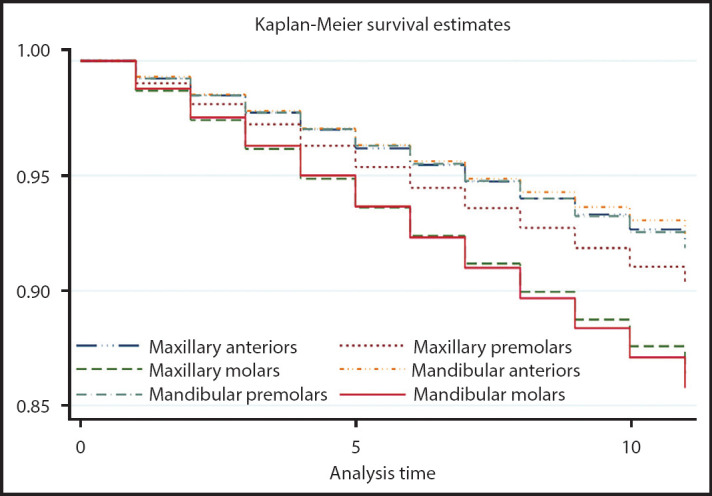
Survival rate of RCT by all tooth type (2002-2013) RCT: Root canal treatment

**Figure 2.**
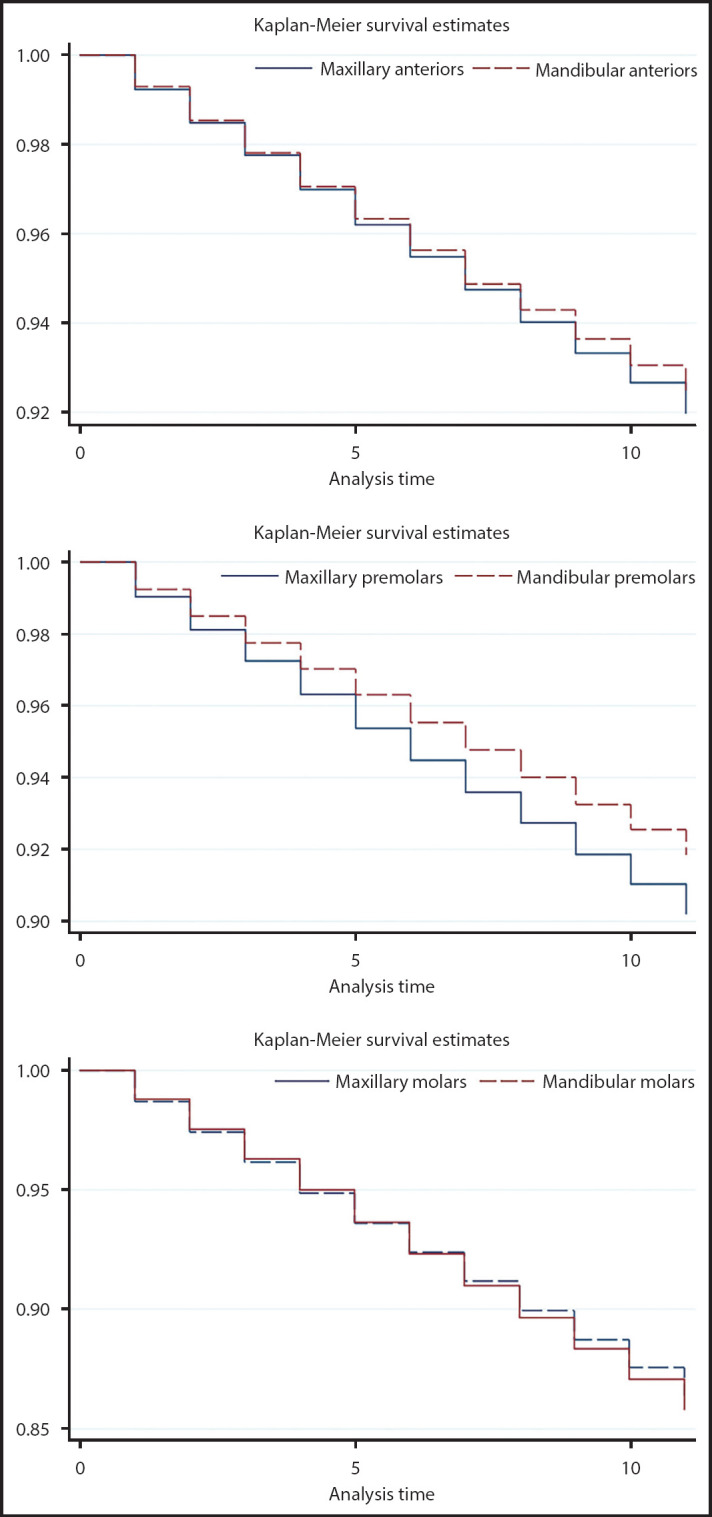
Survival rate of RCT- by type of tooth (2002-2013) RCT: Root canal treatment

The analysis of the survival rate trend that occurred during the observation period (January 1, 2002-December 31, 2013) using the K-M method showed that failure rate of the mandibular molars was higher than that of the maxillary molars, and the mandibular premolars showed a higher survival rate than the maxillary premolars (Fig. 1). Maxillary molars accounted for 32.06% of teeth that had an untoward event, with the second highest percentage after the mandibular molar (Table 2, Fig. 2). Examination according to the maxillary/mandibular tooth type revealed that the mandibular molar had a higher failure rate than the maxillary molar, and the mandibular premolar had a higher survival rate than the maxillary premolar (Fig. 2). There was a statistically significant difference in survival rate according to the type of tooth using the log-rank test. Moreover, it was found that the relative hazard of mandibular anteriors was lower than that of molars or premolars (P<0.001, Table 3). 

**TABLE 3.** Log-rank test according to the type of tooth (2002-2013)

Parameters	Observed	Expected	S.E.	Relative hazard	x^2^	P
Maxillary anteriors	13,801	20324.11	0.027	0.901	10296.73	0.000
Maxillary premolar	20,532	24531.29	0.022	0.865		
Maxillary molar	52,781	44604.58	0.014	1.223		
Mandibular anteriors	5,174	8132.67	0.044	0.657		
Mandibular premolar	13,320	19319.04	0.028	0.712		
Mandibular molar	58,822	47518.3	0.013	1.279		

S.E.: Standard error

Table 4 shows the results of the analysis of the hazard ratio using multivariate Cox regression models to examine the factors affecting the survival rate after RCT. The factors affecting the survival rate after RCT were gender, age, income, health insurance subscription type, type of visits, and tooth type. The hazard ratio for females was 0.70 times that for males, showing that females are less likely to have untoward events after RCT than males. Hazard ratio was significantly higher in participants aged 30-49 years, 50-64 years, and over 65 years; 1.71, 2.56, and 2.95 times, respectively, as compared to participants in their 20’s. 

**TABLE 4.** Effects of patient factors, type of tooth, treatment visits on extraction adjusted using Cox regression analysis

Parameters	Hazard ratio^a^	Standard error	P	95% confidence interval
Gender				
Male	ref.			
Female	0.704	0.004	0.000	0.693-0.714
Age (years)				
20-29	ref.			
30-49	1.719	0.016	0.000	1.671-1.767
50-64	2.563	0.024	0.000	2.490-2.638
≧65	2.959	0.027	0.000	2.864-3.058
Household income quantile				
Low	ref.			
Highest	0.934	0.009	0.001	0.910-0.958
High-middle	0.950	0.010	0.006	0.924-0.977
High	0.947	0.009	0.000	0.922-0.972
Low-middle	0.976	0.010	0.108	0.949-1.005
Lowest (Medical beneficiary)	0.995	0.033	0.937	0.907-1.090
Health insurance subscription type				
Local subscribers	ref.			
Employee	0.952	0.003	0.000	0.945-0.960
Institution type				
Local clinic	ref.			
Hospital	1.054	0.020	0.353	0.993-1.120
Tertiary Hospital	0.949	0.037	0.300	0.849-1.061
Number of visits				
Single-visit for treatment	ref.			
Multiple visits for treatment	0.802	0.006	0.000	0.788-0.816
Tooth type				
Mandibular molar	ref.			
Mandibular premolar	0.954	0.006	0.000	0.937-0.972
Mandibular anteriors	0.674	0.006	0.000	0.658-0.691
Maxillary molar	0.514	0.006	0.000	0.499-0.530
Maxillary premolar	0.506	0.005	0.000	0.491-0.521
Maxillary anteriors	0.440	0.007	0.000	0.420-0.460

^a^Hazard ratio is the relative risk of having adverse reaction when compared with the reference group=1. A hazard ratio >1 indicates a higher risk of developing an adverse outcome (i.e., loss of tooth) relative to the reference group=1

Compared to the first category of income, highest category of income, high-middle category of the income, high category of the income had significantly lower hazard ratio; 0.93 times, 0.95 times, and 0.94 times, respectively. Multiple visits for treatment had 0.80 times lower hazard ratio than single-visit for treatment. According to the tooth type, all the teeth had significantly lower hazard ratio than the mandibular molar. In particular, the maxillary anteriors showed the lowest proportional risk compared to other tooth types. In particular, the risk of untoward events in maxillary anteriors was found to be 0.440 times that of the mandibular molar.

## DISCUSSION

The survival rate of the RCT-treated teeth in the previous studies focused on the clinical factors of RCT, with most studies being based on a small number of research participants. Therefore, to overcome the limitations of these existing studies, this study was conducted to determine the cumulative survival rate of RCT treated teeth in adult patients who underwent RCT using a customized cohort untoward event from the data of the KNHIS. The study also examined the factors affecting the cumulative survival rate by focusing on the participants’ dental healthcare as well as demographic and socioeconomic factors.

There are differences in the perspectives on survival and success as to what constitutes successful treatment in RCT. Therefore, Strindberg presented a strict criterion for the success rate of RCT, which requires that the tooth be asymptomatic with no apical radiation after RCT (13). However, in a study showing 91% of patients were asymptomatic and had no functional problems, the success rate was reduced to 83% on applying these results to Strindberg's criteria (14). However, the criterion for survival rates is less stringent than the criteria for success rates of RCT, as seen in the results of several studies (4, 5, 10, 12). The survival criterion is defined as the treated tooth being asymptomatic after treatment. Successful treatment can be determined by the survival of the tooth. Asymptomatic and functional teeth, regardless of radiological appearance, can be regarded as successful RCT.

In this study, the cumulative survival rate of RCT at 11 years was estimated to be 88.37%. According to a study by Chen et al. (19) using Taiwan's health insurance data to confirm the survival rate of RCT, the 5-year survival rate was 89.7%. In a study using British National Health Service (NHS) data (21), the survival rate for 10 years and 2 years were 74% and 92%, respectively. These differences are expected to vary depending on the variables that can be seen through epidemiological data, the number of samples, the difference in period, and the criteria for success and failure (2, 3, 5, 19). Depending on the type of tooth, RCT of the mandibular molar was found to be performed at a higher frequency, which can be attributed to the fact that the mandibular first molars erupt first and are exposed to the causative factors of caries in the oral cavity for a long period of time (22). The mandibular molar had the lowest survival rate, due to periodontal status or age (3). However, this may be due to the higher probability of occurrence of missed canals during treatment, isthmus, crack, and so on (3, 23). 

Confirmation of the factors that influence the cumulative survival rate of RCT revealed that gender, age, income, health insurance subscription type, number of visits, and the type of tooth were significant influencing factors. The proportional risk of females was lower than that of males. These findings were similar to those of the study by Pineda et al. (12) where male subjects were more likely to undergo tooth extraction after RCT than female subjects. It can be interpreted that this was due to the female subjects being more concerned about oral health than men, and their oral health behaviour being better (11, 24, 25).

Age was also identified as a risk factor for the cumulative survival rate of RCT, and the risk of failure of other groups was significantly higher than that of the 20-29 years age group. Compared to the younger age group, the risk of failure among the middle-aged and the elderly groups was significantly higher. These results were contrary to the results reported in the study of Dummer et al. (26) that the lower the age, the higher the likelihood of RCT failure, and the higher the age, the better the endodontic prognosis. According to the study by Bamise et al. (10), as age increases, RCT due to apical lesions, prostheses, and tooth cracks tend to increase. In this study, the rate of RCT was significantly higher as the age increased. This result could be due to the healing capacity of middle-aged or elderly people decreasing with the passage of time, and the space occupied by the nervous tissue being narrowed and the space being replaced by dentin, which increases the proportional risk (9). 

Income also influenced the survival rate. The proportional risk of the income quartile was lower than that of the first category (low-income group). Even after adjustment for factors such as the demographic characteristics of the patient, tooth type, number of visits, and institution type, the proportional hazard of other groups was lower than that of the first categories (low-income group). These results are meaningful results supporting studies that reported differences in the level of dental care and oral health according to income level. There are no studies correlating the survival rate of RCT with income. Thus, direct research is difficult. However, high self-pay for dental care is still a major barrier to access to dental care. Low-income groups have less poor oral health (11). In conclusion, the results of this study reflect the results of the interest of the high-income group in oral health, their efforts to preserve their teeth, and attention to preventive treatment (11).

In summary, it was confirmed that clinical factors were important with regard to the period for which natural teeth can be maintained after RCT, but socioeconomic factors can also have an effect. Multiple visits for treatment showed a lower proportional risk, which means that teeth with compressed root canal filling are less likely to undergo extraction than those with simple root canal filling (2, 27, 28).

Regarding the type of tooth, the proportional hazards of other teeth were significantly lower than that of the mandibular molar. The order of teeth with a high probability of an untoward event occurring is as follows: mandibular molar, mandibular premolar, mandibular anteriors, maxillary molar, maxillary premolar, and maxillary anteriors. In particular, the high risk of RCT failure in the mandibular molar was also noted by Ricucci et al. (29). This is due to the complex anatomical characteristics of the mandibular molar. Since the mesial root canal of the mandibular molar has a relatively large curvature than other teeth, the possibility of file fracture is high during RCT. Thus, the probability of procedural errors occurring during RCT of the mandibular molar is relatively higher than that of other teeth. In addition, the probability of tooth fracture may vary depending on the tooth shape, which could be interpreted as the inherent weakness of the tooth and the occlusal load differs depending on the tooth shape after RCT (28).

Using representative national data, it was confirmed that factors affecting the survival rate of RCT included gender, age, income, health insurance subscription type, tooth type, and number of visits.

However, there are some limitations to this study. The information on medical records cannot be accurately grasped due to the nature of the NHIS request data used in the study. In addition, factors such as information on non-indemnity treatment and opinions of dental professionals were not sufficiently considered. Therefore, it is expected that in future studies, better research results on factors affecting the survival rate of RCT will be derived by complementing the characteristics of data by linking various data sources such as medical record data and non-payment data. Despite these limitations, this study was meaningful as it was a representative study using data from the NHIS which confirmed the mutual relationship between RCT and patient factors apart from the clinical factors examined in the previous studies.

The findings of this study may be used to assess the survival rate following traditional RCT, using population data. Further research is required to analyze the influence of clinical factors such as rubber dam installation, the presence of crown or full-coverage restoration, and the use of radiographs.

## CONCLUSION

Our study confirmed that the survival rate of RCT after 11 years was 88.37% using the Korean cohort data. In addition, it was confirmed through real-world data that gender, age, income, health insurance subscription type, number of visits, and type of tooth are factors that influence the survival rate of RCT in South Korea. 
